# MicroRNA Regulation of Brown Adipogenesis and Thermogenic Energy Expenditure

**DOI:** 10.3389/fendo.2017.00205

**Published:** 2017-08-23

**Authors:** Farnaz Shamsi, Hongbin Zhang, Yu-Hua Tseng

**Affiliations:** ^1^Section on Integrative Physiology and Metabolism, Joslin Diabetes Center, Harvard Medical School, Boston, MA, United States; ^2^Department of Biomedical Sciences, University of Copenhagen, Copenhagen, Denmark; ^3^Harvard Stem Cell Institute, Harvard University, Cambridge, MA, United States

**Keywords:** microRNA, adipose tissue, brown, non-coding RNAs, uncoupling protein 1, adipogenesis, gene expression regulation

## Abstract

Obesity, diabetes, and associated metabolic diseases have become global epidemics. Obesity results from excess accumulation of white fat, while brown and its related beige fat function to dissipate energy as heat, thus counteracting obesity and its related metabolic disorders. Understanding the regulatory mechanisms for both white and brown adipogenesis provides new insights for prevention and treatment of these metabolic diseases. In addition to traditional gene transcription and translation, microRNA (miRNA) represents a new layer of regulatory mechanism in many biological processes and has attracted a great deal of research interests in exploring their roles in physiological and pathophysiological conditions. This review focuses on the recent advances of regulating brown adipogenesis and energy metabolism by miRNAs, aiming to delineate the regulatory principles of miRNAs on this unique aspect of energy homeostasis.

## Introduction

Obesity has become a global epidemic and major contributor to metabolic syndrome and disorders such as type 2 diabetes, cardiovascular disease, Alzheimer’s disease, and many cancers. There are two types of adipose tissues in human body: white adipose tissue (WAT) and brown adipose tissue (BAT). WAT is specialized to store excess energy in the form of triglycerides and plays a pivotal role in the regulation of energy homeostasis. In addition, WAT is the biggest endocrine organ in the body and secretes several adipocyte-derived hormones, such as adiponectin (1), leptin (1, 2), resistin (3), and others, which regulate insulin sensitivity, appetite, glucose, and lipid metabolism. BAT, on the other hand, is the key site for non-shivering thermogenesis and has a unique capacity to dissipate excess fuel energy as heat. BAT has high mitochondrial density and expresses uncoupling protein 1 (UCP1). UCP1 is a proton channel, localized to the inner mitochondrial membrane that allows protons in the mitochondrial intermembrane space to reenter the mitochondrial matrix without generating ATP. BAT-mediated thermogenesis plays a crucial role in thermostatic regulation, particularly when facing environmental changes such as cold and diet. In addition, BAT possesses an enormous capacity for glucose uptake and plays an important function in both lipid and glucose metabolism (4). Functional BAT has long thought to exist only in newborns but was recently rediscovered in adult humans (5–7). In addition to classical BAT, cold exposure, exercise training and other types of stimulation induce the formation of a type of brown-like adipocytes (known as beige or brite adipocytes) within WAT (a process called “browning” or “beiging” of WAT) (8, 9), which expresses UCP1 and has thermogenic capacity comparable to BAT. Because BAT counteracts energy storage in WAT by promoting energy expenditure, enhancing the development and activity of BAT and beige fat has become an attractive potential strategy to prevent and treat human obesity.

## Developmental Origin of Brown and Beige Fat

Adipocyte differentiation is a coordinated process regulated by a series of transcriptional cascades consisting of both positive and negative regulators. In addition to general adipogenic regulators, brown adipocytes require specific brown fat lineage commitment factors. All these regulators are expressed in a temporal manner along with brown adipocyte differentiation process. Classical interscapular BAT is developed from precursors in the embryonic mesoderm that give rise to both BAT and skeletal muscle cells. These multipotent precursor cells express transcription factors such as paired box 7 (10), engrailed-1 (11), and myogenic factor 5 (MYF5) (12). Lineage tracing experiments have shown that the majority of beige adipocytes in the subcutaneous white fat depot (scWAT) come from a developmentally distinct lineage that lack MYF5 expression (12).

Transition of adipocyte precursors (preadipocytes) to mature adipocytes is orchestrated by a cascade of transcription regulators such as peroxisome proliferator-activated receptor gamma (PPARγ) and members of the CCAAT/enhancer-binding protein family (C/EBPs) (13). In addition to general regulators of adipogenesis essential for both brown and white adipocyte development, the expression of thermogenic gene program in brown/beige adipocytes is regulated by additional transcriptional regulators such as peroxisome proliferative activated receptor gamma coactivator 1 alpha (14), PR domain containing 16 (PRDM16) (15), forkhead box C2 (16), and others.

## MicroRNAs (miRNAs) Roles in Fine-Tuning of Gene Expression

MicroRNAs are a class of short non-coding RNAs consisting of 22 nucleotides and represent a new layer of fundamental regulatory mechanism for transcription and translation (17). miRNAs are key regulators of diverse biological processes, such as proliferation and differentiation and are also involved in the pathophysiology of many diseases (18). In general, miRNAs function as negative regulators of gene expression. They are usually transcribed by RNA polymerase II as primary miRNAs (pri-miRNAs), and then processed by Drosha RNAse III endonuclease and microprocessor complex subunit DGCR8 to generate precursor miRNAs (pre-miRNAs) that are about 60–70 nucleotides long. Following Exportin-5-mediated transport to the cytoplasm, pre-miRNAs are cleaved by a Dicer complex to generate the miRNA/miRNA* double strand. Eventually, mature miRNAs will be loaded into the miRNA-induced silencing complex where they target and bind to the 3′ UTR of specific genes, leading either to mRNA degradation or translational repression (19).

**Figure 1:**
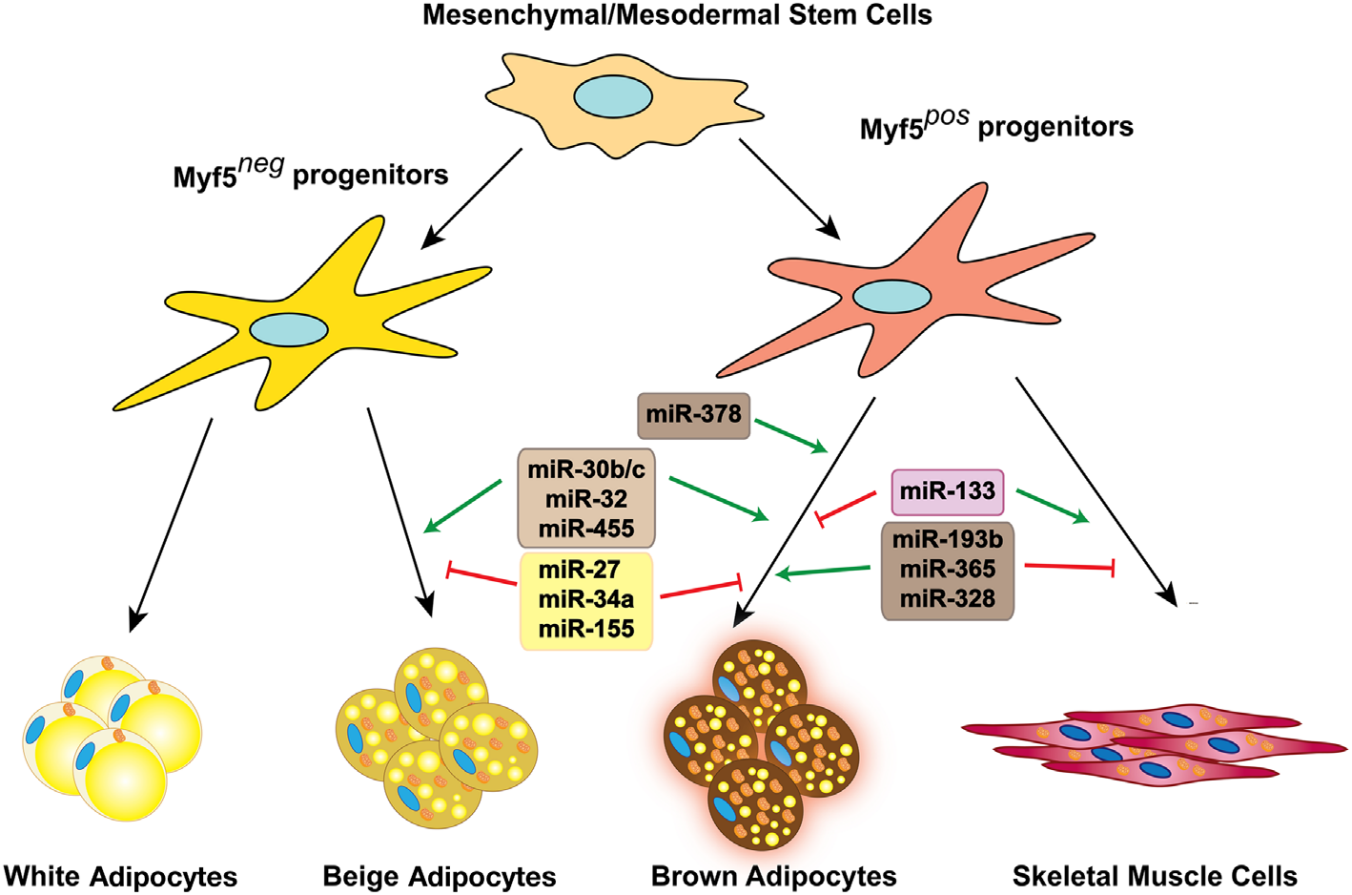
miRNAs involved in brown and beige fat development and function. Several miRNAs positively or negatively regulate brown fat lineage determination and differentiation, as well as beige fat development.

Over the past few years, the role of miRNAs in the regulation of different biological processes has become evident. Numerous studies have pointed to the significance of miRNAs in the regulation of adipose tissue development and function. In this review, we discuss the multiple roles of miRNAs in brown and beige fat biology (Figure 1). In addition, we discuss the newly discovered function of secreted miRNAs as metabolic messengers, enabling communication between different tissues and organs in the body.

Normal miRNA processing has been shown to be essential for the maintenance of brown and WAT function. Aging results in a global downregulation of mature miRNAs in WAT, which is attributed to decreased expression of miRNA processing enzymes, mainly Dicer. This massive loss of miRNAs could be largely prevented by caloric restriction, which is known to expand lifespan in mice and many other species by up to 60% (20). Mice lacking Dicer expression in adipose tissue displayed an abnormal fat accumulation in the interscapular fat depot and decreased mass of the subcutaneous and intra-abdominal fat depots, resembling HIV-associated lipodystrophy phenotype. This was accompanied by whitening of BAT and resulted in severe insulin resistance and dyslipidemia (21, 22). Consistently, partial reduction of Dicer expression in BAT, by deleting only one copy of the gene, impaired BAT thermogenic function and exacerbated the effects of high fat diet-induced obesity on glucose metabolism (23). These observations clearly showed that Dicer function is essential for development of WAT and maintaining BAT identity and function. However, recent studies have provided strong evidence for miRNA-independent roles of Dicer (24, 25). The earliest hints supporting this came from studies showing different phenotypes of Dicer- and Drosha-deficient cells. Therefore, to precisely elucidate the significance of miRNA biogenesis in adipose tissue development, additional animal models lacking other key miRNA processing enzymes would be crucial.

## miRNAs as Activators of Brown Adipogenesis

### miR-193b-365 Cluster

miR-193b and miR-365 are BAT-enriched miRNAs conserved in both humans and mice (26). In mice, they are located within a 5-kb distance on chromosome 16 and are transcribed as a bicistronic pri-miRNA (27). Chromatin immunoprecipitation experiments showed the binding of PPARα to the PPARα/RXR binding elements on the promoter region of this cluster. Blocking miR-193a/b and miR-365 inhibited brown adipocyte differentiation *in vitro* and resulted in a marked reduction in expression of key adipogenic genes including adiponectin, Cebpα, Fabp4, and Pparγ, which is accompanied by a greater decrease in thermogenic genes such as Ucp1, Pparα, Ppargc1α, Dio2, Prdm16, and Cidea. These data suggest that miR-193b and miR-365 function is required for general adipogenesis pathways in addition to playing a role in development of brown adipocytes. miR-193b promotes induction of adipogenic versus myogenic fate, possibly by direct targeting and suppression of an adipogenesis inhibitor, Runx1t1, and two pro-myogenic genes, Cdon and Igfbp5. The physiological significance of these observations was later challenged by *in vivo* studies demonstrating the normal development and function of BAT in miR-193b KO mice. The discrepancy between the *in vitro* and *in vivo* findings might be partially explained by the compensatory downregulation of miR-133a, which acts as an inhibitor of brown adipogenesis (28) in BAT of miR-193b KO mice.

### miR-328

To search for the individual miRNAs that regulate aging and obesity-associated decline in BAT function, Oliverio et al. integrated the expression of miRNAs in a mouse model of premature aging, long-lived Ames dwarf mutants, and diet-induced obese (DIO) mice. They identified miR-328 as a possible regulator of BAT function as its expression was induced in the BAT of the Ames dwarf mice and was decreased in the BAT of aging and DIO models (23). Loss of miR-328 resulted in the downregulation of BAT-selective genes such as Ucp1, Prdm16, Ppargc1α, Cidea, and Cebpα. Interestingly, the seed sequence of miR-328 is similar to that of miR-193b, another BAT-enriched miRNA described above, suggesting that they might share their target genes. Similar to miR-193b, miR-328 promotes brown adipocytes’ differentiation, possibly by suppressing the expression of muscle lineage regulators. Mechanistically, miR-193b and miR-328 target Bace1 (beta-site amyloid precursor protein-cleaving enzyme 1), a muscle enriched gene that promotes myogenesis and inhibit brown fat commitment. Consistent with its function as a negative regulator of BAT differentiation and function, Bace1 expression was induced in BAT of DIO mice and reduced by cold exposure. Moreover, inhibition of Bace1 enzymatic activity enhanced the expression of brown adipocyte markers in BAT and subcutaneous WAT.

### miR-378

miR-378 genomic locus is positioned in the sense strand of the first intron of Ppargc1β, which is highly expressed in brown fat and is one of the key transcriptional regulators of mitochondrial biogenesis. Ectopic overexpression of miR-378 under the control of the aP2 promoter leads to expansion of BAT in mice, accompanied by a reduced mass of WAT depots. The BAT expansion is mainly the result of the enhanced differentiation of brown preadipocytes, and not an increase in cell size. In this model, BAT expansion prevented genetic and diet-induced obesity. Phosphodiesterase Pde1b is shown to be a direct target of miR-378 in BAT. miR-378-mediated downregulation of Pde1b enhances brown adipocyte differentiation by regulating cAMP turnover in BAT (29). miRNA profiling of abdominal subcutaneous adipose tissue from gastrointestinal cancer patients with or without cachexia identified upregulation of miR-378 in patients with cachexia. Overexpression of miR-378 catecholamine stimulated lipolysis in human adipocytes, suggesting that it may play a role in elevated lipolysis that results in adipose tissue loss in cancer cachexia (30).

### miR-30b/c

In addition to their function in regulation of adipogenesis (31), members of the miR-30 family have been shown to play a role in regulation of the thermogenic gene program in brown and beige fat (32). miR-30b is expressed in both human and mouse BAT (26). miR-30b and miR-30c expression is induced in BAT and subcutaneous WAT in response to cold exposure, β3-adrenergic receptor activator CL-316,243, non-selective β-adrenergic receptor activator isoproterenol or the cAMP inducer forskolin. Overexpression of miR-30b and miR-30c induced Ucp1 expression in BAT and subcutaneous WAT, both *in vitro* and *in vivo*. The positive effect of miR-30b/c on UCP1 expression seems to be mediated by targeting receptor-interacting protein 140 (Rip140). Rip140 is a transcriptional corepressor for nuclear receptors and is involved in the silencing of Ucp1 expression in white adipocytes through recruitment of chromatin remodeling enzymes, which enhance histone deacetylation and methylation of the Ucp1 enhancer and promoter (33).

### miR-455

miR-455 was identified as a marker of BAT, acting downstream of BMP7 and cold-induced pathways to promote brown adipocyte lineage commitment (34). In both rodents and humans, miR-455 is selectively expressed at higher levels in BAT compared with WAT, miR-455 overexpression promotes brown adipocyte differentiation in committed brown and white preadipocytes and in non-committed multipotent progenitor cells *in vitro* by inducing the expression of key regulators of adipogenesis such as Pparγ, Cebpα, and Cebpδ, and important brown adipocyte markers Ucp1, Ppargc1α, Prdm16, and Cidea. Consistent with the *in vitro* findings, mice overexpressing miR-455 in adipose tissue using an aP2 promoter-driven transgene (FAT455 mice) exhibited an enhanced thermogenic capacity in response to cold or norepinephrine stimulation, which resulted from elevated Ucp1 expression in both BAT and subcutaneous WAT depots. As commonly seen in mouse models with higher energy expenditure, FAT455 mice showed an increase in food consumption. Under pair-fed condition, FAT455 mice were more resistant to weight gain and had improved insulin sensitivity and glucose tolerance compared to their wild-type littermates. miR-455 directly targets Runx1t1 and Necdin, both of which act as inhibitors of white and brown adipogenesis. In addition, miR-455 targets HIF1an. HIF1an is an asparaginyl hydroxylase enzyme regulating the activities of multiple cellular pathways through hydroxylation of Asn residues in substrates. In brown preadipocytes, HIF1an directly interacts with the AMPKα1 isoform and inhibits AMPK activity, at least in part, *via* asparaginyl hydroxylation. Therefore, miR-455 overexpression suppresses HIF1an expression, leading to removing an inhibitory signal of AMPK and allowing AMPK to exert its full function. Activated AMPK in turn triggers Ppargc1α phosphorylation, which could eventually enhance its transcriptional activity and result in promotion of brown adipogenesis.

### miR-32

miR-32 is a BAT-enriched miRNA, recently identified to be located in close proximity of a BAT specific super enhancer (35). miR-32 expression in BAT is induced by cold exposure, and it regulates the thermogenic gene program. Inhibition of miR-32 in mice using an antisense oligo (ASO) impaired the activation of thermogenic response upon cold challenge and reduced energy expenditure. The compromised thermoregulatory capacity was due to both lower UCP1 induction in BAT and reduced recruitment of beige cells in scWAT. Mice injected with miR-32 ASO failed to upregulate FGF21 expression in BAT in response to cold exposure. Thus, miR-32 inhibition resulted in decreased serum FGF21 levels, which led to reduced browning of scWAT upon cold exposure. Transducer of ErbB-2.1 (Tob1) was identified as a direct target of miR-32. Tob1 repression by miR-32 results in activation of p38 MAP kinase signaling and ATF2 transcription factor to elevate FGF21 expression in BAT. Secreted FGF21 from BAT communicates with scWAT to promote browning of this depot in response to prolonged cold challenge.

## miRNAs as Inhibitors of Brown Adipogenesis

### miR-27

miR-27 negatively regulates white and brown adipogenesis in mice (36–38) and humans (39, 40). Expression of miR-27a and miR-27b was downregulated in BAT and scWAT of mice in response to cold exposure. Inhibition of miR-27 in brown preadipocytes enhanced the expression of multiple key transcription factors including Pparα, Pparγ, Prdm16, and Ppargc1α and facilitated the differentiation toward Ucp1 expressing adipocytes. Conversely, miR-27 overexpression reduces the expression of Prdm16 and Ucp1 during brown and beige adipogenesis. *In vitro* luciferase assay results demonstrated that miR-27 directly targets the 3′ UTR of both Prdm16 and Pparα, suggesting that the inhibitory effect of miR-27 on brown adipogenesis might be through modulation of these factors.

### miR-34a

miR-34a was identified as a negative regulator of brown and beige formation in obese mice. Expression of miR-34a was positively associated with BMI in human (41) and is upregulated in DIO mice (42). Lentiviral-mediated knockdown of miR-34a in DIO mice promotes the browning of multiple white fat depots including perirenal WAT and gonadal WAT, as well as scWAT, and protects mice from the detrimental metabolic effects of high fat diet. Mechanistically, miR-34a targets FGFR1 and therefore attenuates FGF21 responsiveness and downstream signaling in adipose tissue. SIRT1 is another direct target of miR-34a that plays a critical role in transcriptional regulation of several brown and beige fat markers through deacetylation of Ppargc1a. Given that FGF21 signaling has also been linked to modulation of Ppargc1a activity, miR-34a is likely to negatively regulate the expression of browning genes in obesity through suppression of Ppargc1a transcriptional activity. Interestingly, the beneficial effects of miR-34a inhibition go beyond its browning effects, as it also improves FGF21 signaling in the liver, which contributes to improved overall metabolism and decreased adiposity.

### miR-133

miRNA microarray analysis of BAT from mice exposed to cold for 24 h identified miR-133 as one the most downregulated miRNAs after cold exposure. miRNA-133 is highly enriched in cardiac and skeletal muscle lineages. It was shown that miRNA-133 negatively regulates brown adipogenesis through suppression of Prdm16. Inhibition of miRNA-133 in brown and beige preadipocytes increases Prdm16, Pparγ, and Pparα expression and results in the elevation of Ucp1 expression after differentiation. Downregulation of miR-133 by β-adrenergic stimulation was suggested to be mediated by the transcriptional factors from the Mef2 family, Mef2c, Mef2a, and Mef2d (28). miR-133a and miR-133b are also expressed in multipotent satellite cells in adult skeletal muscle and are involved in lineage commitment and fate decision between myogenic and brown adipose lineages, primarily by targeting Prdm16. Inhibition of miR-133 resulted in the formation of metabolically active brown adipocytes in regenerating tibialis anterior muscles, which in turn promotes energy expenditure and protects mice against DIO (43).

### miR-155

miR-155 is highly expressed in preadipocytes isolated from the stromal-vascular fraction of BAT, and its expression is dramatically reduced in differentiated brown adipocytes (44). miR-155 targets adipogenic transcription factor Cebpβ, and therefore suppresses the development of adipogenic and thermogenic programs. Cebpβ also directly binds to the distal site E in the miR-155 regulatory region, and negatively regulates miR-155. Therefore, miR-155 and Cebpβ form a double-negative feedback loop, which ensures efficient induction of Cebpβ upon adipogenic induction. Overexpression of miR-155 in brown preadipocytes suppresses adipogenic differentiation and induction of the thermogenic program in brown adipocytes. Consistently, it was found that miR-155 is a downstream effector of TGFβ1 signaling, which is known to block adipogenesis *in vitro* (45) and *in vivo* (46) through modulating the transcriptional activity of C/EBPs (47). *In vivo* overexpression of miR-155 selectively impaired brown fat development, while WAT was not affected. Conversely, loss of miR-155 enhanced the thermogenic capacity of BAT and resulted in elevated levels of scWAT browning in cold (44).

## BAT as an Endocrine Organ Secreting miRNAs

In addition to the well-known function of BAT in non-shivering thermogenesis and energy expenditure, recent studies have established BAT as an endocrine organ that secretes several “batokine” (48–54) and “lipokine” (55, 56) molecules, which mediate the cross-talk between different tissues in the body. More recently, using the adipose tissue-specific Dicer KO mouse model (ADicer KO), it was shown that brown and WAT contribute a major fraction of circulating exosomal miRNAs. Importantly, elevated FGF21 expression observed in the liver of ADicer KO mice can be lowered by the injection of exosomes from the serum of wild-type donors. Adenoviral delivery of hsa-miR-302f to the BAT of mice resulted in suppression of a miR-302f 3′ UTR reporter in liver (57). These observations suggest that BAT-derived miRNAs modulate gene expression in the liver, and potentially in other organs, as well.

miR-99b level was strongly reduced in circulating exosomes of ADicer KO mice and was restored by BAT transplantation. Treatment with exosomes from ADicer KO mice reconstituted with miR-99b dramatically reduced FGF21 expression in liver, indicating that adipose tissue-derived exosomal miR-99b regulates FGF21 expression in liver.

**Figure 2:**
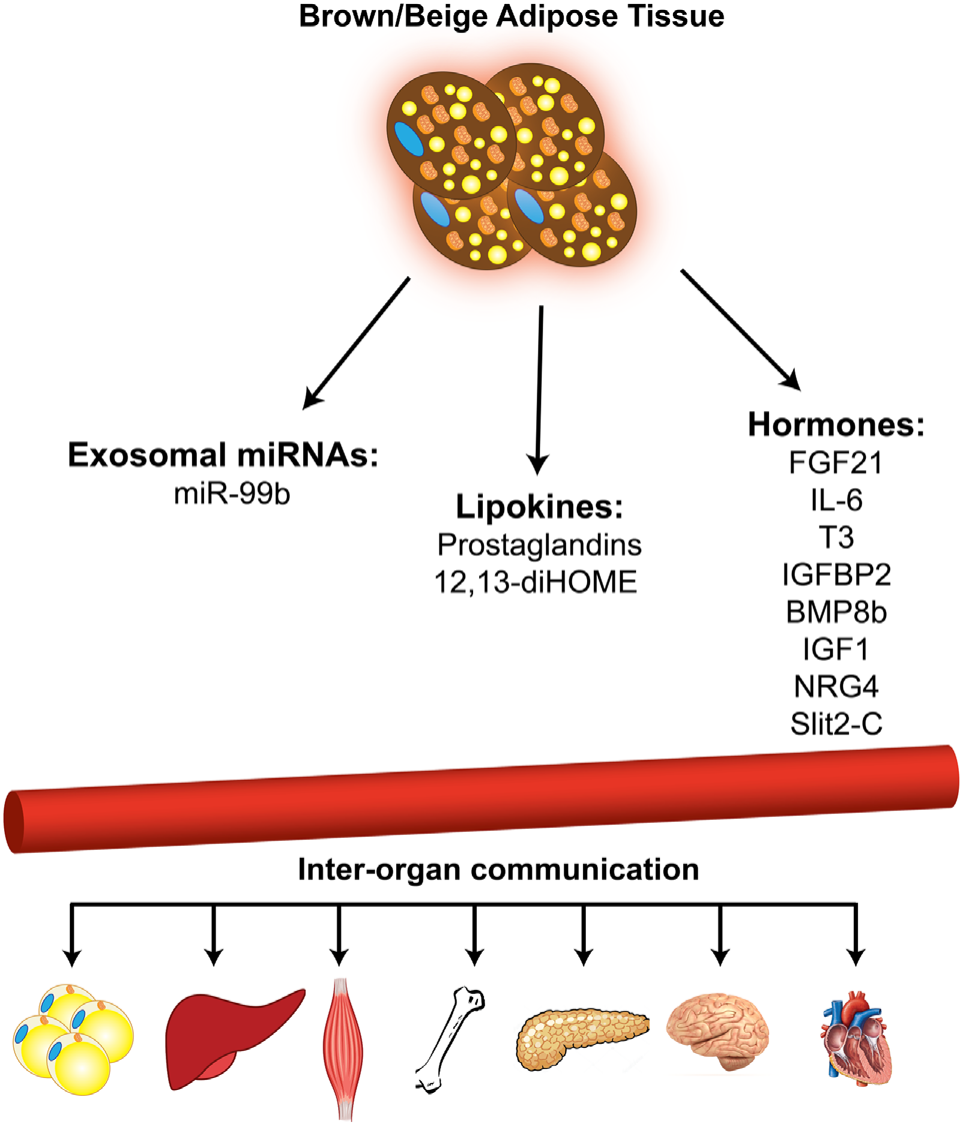
Brown and beige adipose tissues function as secretory organ. Brown and beige adipose tissues communicate with other metabolic organs through secretion of miRNAs, lipid molecules, and hormones.

It has been shown that *in vitro* and *in vivo* activation of brown adipocytes results in increased release of exosomes (58). Exosomes released by cold-activated brown fat displayed a unique miRNA profile with higher levels of miR-34c* and lower levels of miR-92a compared with the brown fat of control mice maintained at room temperature. Interestingly, cold acclimation in humans lowered the exosomal miR-92a abundance in serum, and the level of miR-92a in human serum was shown to be inversely correlated with BAT activity. Based on this, exosomal miR-92a might serve as a biomarker for BAT activity in humans (Figure 2).

## Concluding Remarks

Obesity is a major contributor to metabolic syndrome and disorders such as type 2 diabetes and cardiovascular diseases. Rediscovery of functional brown fat in adult humans has opened new avenues for utilizing its prominent capacity for fuel consumption and energy expenditure to combat obesity and its comorbidities (5–7). Investigation of therapeutic approaches focusing on BAT and its thermogenic capacity has gained considerable interest in the last several years. In addition to improving energy balance, activated BAT improves insulin sensitivity in both humans and mice (59, 60).

Due to their key roles in the regulation of gene expression networks, miRNAs are now considered a novel class of therapeutic targets. The recent trials for the application of miRNA mimics or inhibitors as drugs in humans have strengthened the idea of using miRNA-based therapeutics in humans for a range of diseases (61, 62). Some of the associated challenges that need to be overcome before establishing miRNA-based drugs include optimizing the tissue-specific delivery methods, characterizing and minimizing the off-target effects, and evaluating the toxicity and immunological responses. Therefore, we foresee that in the near future antagonizing or restoring specific miRNAs will be used as novel therapeutic strategies to target human BAT and WAT, and such therapies will benefit metabolism by improve energy expenditure and fuel homeostasis.

## Author Contributions

FS and Y-HT wrote the manuscript. HZ provided help and feedback.

## Conflict of Interest Statement

The authors declare that the research was conducted in the absence of any commercial or financial relationships that could be construed as a potential conflict of interest.
